# Application of the 2012 Systemic Lupus International Collaborating Clinics classification criteria to patients in a regional Swedish systemic lupus erythematosus register

**DOI:** 10.1186/s13075-015-0521-9

**Published:** 2015-01-10

**Authors:** Anna Ighe, Örjan Dahlström, Thomas Skogh, Christopher Sjöwall

**Affiliations:** Rheumatology/AIR, Department of Clinical and Experimental Medicine, Linköping University, University Hospital, SE-581 85 Linköping, Sweden; Swedish Institute for Disability Research, Department of Behavioural Sciences and Learning, Linköping University, SE-581 83 Linköping, Sweden

## Abstract

**Introduction:**

In 2012, the Systemic Lupus International Collaborating Clinics (SLICC) network presented a new set of criteria (SLICC-12) to classify systemic lupus erythematosus (SLE). The present study is the first to evaluate the performance of SLICC-12 in an adult European study population. Thus, SLICC-12 criteria were applied to confirmed SLE cases in our regional SLE register as well as to individuals with a fair suspicion of systemic autoimmune disease who were referred to rheumatology specialists at our unit.

**Methods:**

We included 243 confirmed SLE patients who met the 1982 American College of Rheumatology (ACR-82) classification criteria and/or the Fries ‘diagnostic principle’ (presence of antinuclear antibodies on at least one occasion plus involvement of at least two defined organ systems) and 55 controls with possible systemic autoimmune disease, including the presence of any SLE-related autoantibody.

**Results:**

SLICC-12 showed a diagnostic sensitivity of 94% (95% confidence interval (CI), 0.90 to 0.96) compared with 90% (95% CI, 0.85 to 0.93) for the updated set of ACR criteria from 1997 (ACR-97), whereas ACR-82 failed to identify every fifth true SLE case. However, the disease specificity of SLICC-12 reached only 74% (95% CI, 0.60 to 0.84) and did not change much when involvement of at least two different organs was required as an indicator of systemic disease. In addition, SLICC-12 misclassified more of the controls compared to ACR-82, ACR-97 and Fries.

**Conclusions:**

Establishing a standard definition of SLE continues to challenge lupus researchers and clinicians. We confirm that SLICC-12 has advantages with regard to diagnostic sensitivity, whereas we found the diagnostic specificity to be surprisingly low. To accomplish increased sensitivity and specificity figures, a combination of criteria sets for clinical SLE studies should be considered.

## Introduction

Symptoms related to systemic lupus erythematosus (SLE) are numerous and highly variable. Whereas common manifestations in joints, skin, mucous membranes, bone marrow and kidneys are usually rather easily identified, more subtle manifestations, such as neurological symptoms, may remain unrecognized or not judged to be related to SLE [1]. The disease course is unpredictable, with episodes of flares and remissions, sometimes leading to permanent organ damage and preterm death [2].

The profound heterogeneity of SLE causes problems regarding diagnostic accuracy in clinical practice and particularly in clinical research. Over the years, great efforts have been made to establish meaningful sets of criteria to scientifically classify and differentiate the many rheumatic diseases. The American College of Rheumatology (ACR) conducted the first SLE criteria in 1971, but these criteria did not gain much attention [3,4]. By contrast, the revised and validated ACR classification criteria of 1982 (ACR-82) have been used extensively, although concerns were raised early that they were too conservative and outmoded [5]. For instance, ACR-82 does not consider lowered complement levels, but includes a positive lupus erythematosus (LE) cell test as well as a positive Wassermann reaction (false-positive serological test for syphilis) as part of the tenth criterion (immunologic disorder), although these tests are in general regarded as obsolete [6]. In addition, ACR-82 does not consider many of the common cutaneous and neurological manifestations found in SLE and does not accept biopsy-proven lupus nephritis (LN) as SLE in the absence of other manifestations. As an alternative, Fries and Holman’s diagnostic principle has been found to be clinically useful. It is based on the presence of antinuclear antibodies (ANAs) on at least one occasion plus signs of systemic disease with involvement of at least two defined organ systems (including skin, joints, kidney, serosa, blood, lungs and nervous system) [7,8]. One approach to modernizing the ACR criteria was made by Hochberg in a letter to *Arthritis & Rheumatism* with the suggestion of removing the LE cell item and replacing it with lupus anticoagulant and anticardiolipin antibodies of the immunoglobulin M (IgM) or IgG class [9]. Unfortunately, validation of the proposed 1997 update of the ACR criteria (ACR-97) was not performed until recently.

The Systemic Lupus International Collaborating Clinics (SLICC) network, devoted to clinical research in SLE, presented a new set of classification criteria in 2012 (SLICC-12) based on the evaluation of almost 1,400 patient scenarios [10]. Their work included both a derivation and a validation of the new set of criteria as well as of ACR-97. The SLICC-12 criteria contain additional clinical and immunological items. Fulfilment of four or more criteria (including at least one clinical and one immunologic item) of the eleven clinical and six immunologic items are required for an SLE diagnosis according to SLICC-12. An important addition is that biopsy-proven LN in combination with the presence of ANAs and/or antibodies to double-stranded DNA (anti-dsDNA) appears as a stand-alone item that is accepted as SLE according to SLICC-12, regardless of other organ manifestations. In the validation set, the SLICC-12 criteria were noted to be more sensitive but less specific than ACR-97, but they also resulted in fewer misclassifications [10]. Although much indicates that the SLICC-12 criteria will contribute to a higher sensitivity for detecting SLE compared with previous classification grounds, it is still unclear how much acceptance they will receive.

The present study was undertaken to apply the SLICC-12 criteria and compare them with ACR-82, ACR-97 and Fries and Holman’s diagnostic principle (hereinafter referred to as ‘Fries’), based on well-characterized SLE cases included in a Swedish regional SLE register as well as on autoantibody-positive individuals referred to our rheumatology unit on the suspicion of systemic autoimmune disease. Inspired by Fries and Holman, we also aimed to further stress the requirements for SLICC-12 by demanding that an SLE diagnosis be based on involvement of at least two different organ systems as an indicator of systemic disease [7].

## Methods

### Confirmed systemic lupus erythematosus cases

A total of 243 patients with established (84%) or recent-onset (16%) SLE (213 women and 30 men; mean age in January 2014 was 52.8 years, ranging from ages 20 to 92) were consecutively recruited to the prospective follow-up programme KLURING (a Swedish acronym for ‘Clinical Lupus Register in Northeastern Gothia’) at the Rheumatology Clinic, Linköping University Hospital, Sweden, between September 2008 and January 2014. The patient material has previously been described in detail [11,12]. The following criteria were used for entry into KLURING: (1) a clinical SLE diagnosis based on a history of abnormal ANA titres and at least two defined organ manifestations at the time of diagnosis (Fries) and/or (2) meeting the ACR-82 criteria [6,7]. Of the 58 patients who met the ACR-82 item for ‘renal disorder’, 48 (83%) had undergone renal biopsy for confirmation of suspected LN. Further characteristics of the patients with SLE are given in Table 1.

**Table 1:** **Characteristics of the 243 included patients with confirmed systemic lupus erythematosus** ^**a**^

**Characteristics**	**All confirmed SLE cases (** ***N*** **= 243)**		**ACR-82 (** ***n*** **= 197)**		**Fries only (** ***n*** **= 46)**		**ACR-82 vs. Fries** ***P*** **-values**
	**Mean**	**(Range)**	**Mean**	**(Range)**	**Mean**	**(Range)**	
Fulfilled ACR-82 criteria, *n*	4.6	(3 to 9)	5.0	(4 to 9)	3.0	(3 to 3)	<0.0001^b^
SLICC/ACR damage index	1.6	(0 to 9)	1.7	(0 to 9)	1.2	(0 to 7)	0.25^b^
Disease duration, yr	15.1	(0 to 52)	15.6	(0 to 52)	12.9	(1 to 40)	0.18^b^
Age, yr	52.8	(20 to 92)	52.2	(20 to 92)	55.6	(20 to 82)	0.20^b^
Female sex, %	87.7		87.5		89.1		0.74^b^
Caucasians, %	92.2		91.4		95.7		0.33^b^
***Clinical manifestations (%)***							
Acute cutaneous lupus	65.8		71.6		41.3		<0.0001^c^
Chronic cutaneous lupus	15.2		17.8		4.3		0.023^c^
Photosensitivity	50.6		56.3		26.1		0.0002^c^
Nonscarring alopecia	21.4		22.3		17.4		0.46^c^
Oral ulcers	11.1		12.7		4.3		0.11^c^
Arthritis	75.7		76.1		73.9		0.75^c^
Pleuritis	37.9		39.1		32.6		0.41^c^
Pericarditis	14.8		15.2		13.0		0.71^c^
Renal disorder	23.9		28.9		2.2		<0.0001^c^
Biopsy-proven lupus nephritis	19.8		23.9		2.2		0.0009^c^
Neurologic disorder (ACR-82)	4.9		5.6		2.2		0.34^c^
Neurologic disorder (SLICC-12)	10.7		12.7		2.2		0.038^c^
Haemolytic anaemia	4.9		6.1		0		0.086^c^
Leukopenia	29.2		33.0		13.0		0.0074^c^
Lymphopenia	29.2		34.0		8.7		0.0007^c^
Thrombocytopenia	11.5		12.7		6.5		0.24^c^
***Immunologic criteria (%)***							
Antinuclear antibody	98.8		98.5		100.0		0.40^c^
Anti-dsDNA	45.3		53.3		10.9		<0.0001^c^
Anti-Sm	8.2		9.1		4.3		0.29^c^
Lupus anticoagulant^d^	36.3		34.3		43.6		0.097^c^
Anti-cardiolipin^e^	28.5		30.7		19.5		0.23^c^
Anti-β_2_-glycoprotein-1^f^	29.2		30.0		25.9		0.80^c^
Low complement	47.3		51.3		30.4		0.011^c^
Direct Coombs test^g^	51.3		58.1		29.6		0.25^c^

^a^ACR-82, American College of Rheumatology 1982 criteria; Anti-dsDNA, Antibodies to double-stranded DNA; Anti-Sm, Anti-Smith antibody; Fries, Fries and Holman’s diagnostic principle [7]; SLE, Systemic lupus erythematosus; SLICC-12, 2012 Systemic Lupus International Collaborating Clinics criteria. ^b^ *P*-values based on Mann–Whitney *U* test. ^c^ *P*-values based on χ^2^ test. Criteria were tested on any occasion in ^d^182 of 243 patients, ^e^207 of 243 patients, ^f^137 of 243 patients and ^g^113 of 243 patients.

### Controls

A total of 55 individuals (48 women and 7 men; mean age in January 2014 of 49.0 years, ranging from ages 18 to 85) who had been referred to the Rheumatology Clinic, Linköping University Hospital on the suspicion of systemic autoimmune disease were included as control cases. All individuals had at least one immunologic abnormality on the basis of any of the following: positive ANA by immunofluorescence microscopy (IF-ANA); or the presence of any of the antibodies against extractable nuclear antigens (ENAs); i.e. SSA, SSB, Sm, small nuclear ribonucleic proteins, Scl-70 or Jo-1; or IgM and/or IgG class antibodies against phospholipid (PL)-related antigens (cardiolipin and/or β_2_-glycoprotein-1) or a positive test for lupus anticoagulant; or signs of plasma complement consumption. Two of the controls had undergone renal biopsy as a confirmation of suspected LN. Further characteristics of the control cases are presented in Tables 1 and 2.

**Table 2:** **Characteristics of the 55 included control cases** ^**a**^

**Characteristics**	**Control cases (** ***n*** **= 55)**	
	**Mean**	**(range)**
Fulfilled ACR-82 criteria, *n*	2.2	(1 to 3)
Age, yr	49.0	(18 to 85)
Female sex, %	87.3	
***Clinical manifestations (%)***		
Acute cutaneous lupus	34.5	
Chronic cutaneous lupus	1.8	
Photosensitivity	30.9	
Nonscarring alopecia	10.9	
Oral ulcers	3.6	
Arthritis	38.2	
Pleuritis	12.7	
Pericarditis	9.1	
Renal disorder	3.6	
Biopsy-proven lupus nephritis	3.6	
Neurologic disorder (ACR-82)	0	
Neurologic disorder (SLICC-12)	3.6	
Haemolytic anaemia	1.8	
Leukopenia	14.5	
Lymphopenia	12.7	
Thrombocytopenia	7.3	
***Immunologic criteria (%)***		
Antinuclear antibody	89.1	
Anti-dsDNA	10.9	
Anti-Sm	0	
Lupus anticoagulant	36.5	
Anti-cardiolipin	21.6	
Anti-β_2_-glycoprotein-1^b^	21.6	
Low complement	14.5	
Direct Coombs test^c^	13.6	

^a^ACR-82, American College of Rheumatology 1982 criteria; Anti-dsDNA, Antibodies to double-stranded DNA; Anti-Sm, Anti-Smith antibody; SLICC-12, 2012 Systemic Lupus International Collaborating Clinics criteria. Criteria were tested on any occasion in ^b^37 of 55 cases and ^c^44 of 55 cases.

### Laboratory analyses

IgG class ANAs were analysed by indirect IF microscopy using multispot slides with fixed HEp-2 cells (ImmunoConcepts, Sacramento, CA, USA) as previously described [11]. The cutoff level for a positive ANA test was set at a serum dilution of 1:200, corresponding to above the 95th percentile among 150 healthy female blood donors. Microscope slides with fixed *Crithidia luciliae* (ImmunoConcepts) and fluorescein isothiocyanate (FITC)–conjugated γ-chain-specific anti-human IgG as the detection antibody (DAKO, Glostrup, Denmark) were used to analyse IgG class anti-dsDNA antibodies by IF microscopy with cutoff at serum dilution 1:10, corresponding to above the 99th percentile among 100 healthy blood donors (50 women, 50 men).

Antibodies to ENA were screened using a line-blot technique as previously described [11]. To qualify as a positive test for anti-Sm antibody, confirmation by double radial immunodiffusion in gel (ImmunoConcepts) was always required. Lupus anticoagulant was performed by the dilute Russell’s viper venom test. Detection of antibodies against cardiolipin and β_2_-glycoprotein-1 were performed using an enzyme-linked immunosorbent assay (Orgentec Diagnostika, Mainz, Germany) and/or by a fluorescence immunoassay (ELiA; Phadia, Uppsala, Sweden). Only IgM and IgG class antibodies against cardiolipin and β_2_-glycoprotein-1 (as included in the ACR-97 criteria) were analysed, whereas analysis of IgA (as included in the SLICC-12 criteria) was not performed. Levels of complement proteins C3 and C4 were measured by nephelometry in fresh frozen plasma samples, and classical complement function was assessed by a haemolytic assay. The direct Coombs test was analysed by haemolysis in gel (BIO-RAD Laboratories, Hercules, CA, USA).

In each patient for whom laboratory abnormalities were crucial to determining which set(s) of criteria were met, all analyses regarding immunologic criteria were analysed (further specified in Tables 1 and 2).

### Statistics

Clinical and immunologic criteria were described by their proportions (%). Differences between cases meeting ACR-82 and Fries only were examined by Mann–Whitney *U* tests or χ^2^ tests of homogeneity. Classifications of participants based on Fries, ACR-82, ACR-97, SLICC-12 and SLICC-12 with a requirement for involvement of at least two organ systems for SLE diagnosis (SLICC-12:2), separately and in combinations, were examined. Sensitivity and specificity figures were calculated for the confirmed cases (*n* = 243) and the controls (*n* = 55). Positive predictive value (PPV) and negative predictive value (NPV) were calculated as the average PPV and NPV based on a procedure where the controls (*n* = 55) were combined 1 million times with a random sample of the same size (*n* = 55) from the confirmed cases. The Wilson score method was used to calculate 95% confidence intervals (CIs) [13].

### Ethical considerations

Oral and written informed consent was obtained from all subjects. The study protocol was approved by the Linköping University Ethical Review Board, Sweden (M75-08/2008).

## Results

As indicated in Table 1, 197 (81%) of 243 confirmed SLE cases fulfilled the ACR-82 criteria, whereas 46 (19%) met the Fries criteria only. The presence of either IgM or IgG class antibodies against cardiolipin or β_2_-glycoprotein-1 and/or a positive lupus anticoagulant test (not classified as an immunologic criterion according to ACR-82) was found in 23 (50%) of the cases meeting the Fries criteria alone. Of the 243 confirmed cases, 220 (91%) fulfilled the ACR-97 criteria.

In addition to the number of fulfilled ACR-82 criteria, significant differences between the ACR-82 and Fries groups were found for certain manifestations related to skin, renal, neurologic, hematologic and immunologic items. On average, the 55 controls met significantly fewer ACR-82 criteria than the confirmed cases (*P* < 0.0001). The slightly younger average age of the controls (49 years; age range, 18 to 85) compared to confirmed cases (52.8 years; age range, 20 to 92) was not statistically significant (*P* = 0.14). Also, the female predominance was similar among cases (87.7%) and controls (87.3%).

**Figure 1:**
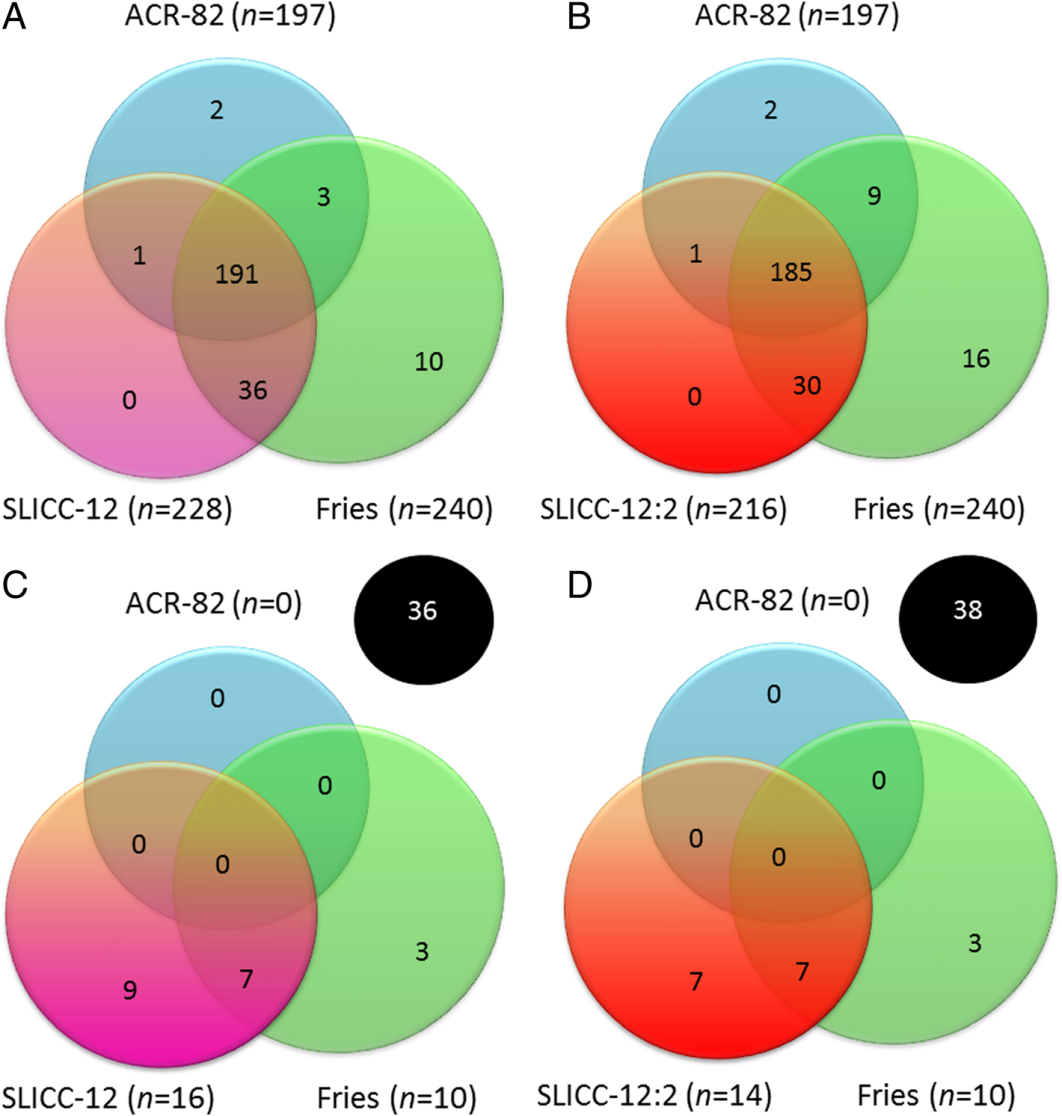
**Venn diagrams illustrating the distribution of confirmed systemic lupus erythematosus cases and controls for each separate classification ground.** Venn diagrams demonstrate the distribution of the 243 confirmed (SLE) cases identified **(A)** by the 1982 American College of Rheumatology (ACR-82) criteria (blue), Fries (green) and SLICC-12 (pink), and **(B)** by ACR-82 criteria (blue), Fries and Holman diagnostic principle (Fries [7]) (green) and the 2012 Systemic Lupus International Collaborating Clinics criteria with SLICC-12 with a requirement for involvement of at least two organ systems for SLE diagnosis (SLICC-12:2) (red) (that is, with the requirement to involve at least two different organ systems). The 55 controls identified **(C)** by ACR-82 (blue), Fries (green) and SLICC-12 (pink) and **(D)** by ACR-82 (blue), Fries (green) and SLICC-12:2 (red). Controls who did not meet any of the classification grounds are indicated in black.

As demonstrated in Figure 1A, SLICC-12 identified 228 (94%) of 243 confirmed cases. To emphasize the systemic nature of SLE, we further increased the requirements for SLICC-12 by demanding involvement of at least two organ systems for SLE diagnosis (SLICC-12:2), but still with the exception of biopsy-proven LN (in combination with ANA/anti-dsDNA), which was considered a stand-alone item. As illustrated in Figure 1B, SLICC-12:2 identified 216 (89%) of 243 confirmed cases. Thus, 12 confirmed cases identified by SLICC-12 (5.2%) had involvement of only one organ system together with fulfilment of immunologic criteria.

The 55 controls (Table 2) eventually received the following diagnoses: undifferentiated connective tissue disease (UCTD) (*n* = 13) [14]; primary Sjögren’s syndrome (*n* = 10); antiphospholipid syndrome (APS) (*n* = 7); rheumatoid arthritis (RA) (*n* = 6); arthralgia (*n* = 4); fibromyalgia (*n* = 3); SLE (*n* = 2); adult-onset Still’s disease (*n* = 1); polymyositis (*n* = 1); systemic sclerosis with primary biliary cirrhosis (*n* = 1); mixed connective tissue disease (*n* = 1); pyogenic arthritis, pyoderma gangrenosum and acne syndrome (*n* = 1); renal infarction (*n* = 1); multiple sclerosis (*n* = 1); psoriatic arthritis (*n* = 1); unspecified arthritis (*n* = 1); and recurrent pleuritis (*n* = 1).

The Fries, ACR-82 and ACR-97 criteria all failed to identify the two true SLE cases with biopsy-proven LN amongst the controls, whereas these cases were captured by the SLICC-12 criteria (Figure 1C). On the other hand, SLICC-12 incorrectly classified 14, Fries 10 and ACR-97 4 non-SLE cases as SLE. These misclassified cases were primarily patients deemed to have APS, RA, UCTD or primary Sjögren’s syndrome. On the basis of SLICC-12:2, 12 of the controls still were misclassified as SLE (Figure 1D).

On the basis of combined data from the confirmed cases and controls (*n* = 298), sensitivity, specificity, PPVs and NPVs were calculated for each separate classification ground, as well as for some combinations (Table 3). ACR-82 was found to have very high disease specificity, whereas the sensitivity was 80% (95% CI, 0.75 to 0.85). ACR-97 reached both sensitivity and specificity of around 90%. Although the sensitivity was even higher with SLICC-12, at 94% (95% CI 0.90 to 0.96), the specificity was low, at 74% (95% CI, 0.60 to 0.84), and did not increase appreciably with the use of SLICC-12:2 (77%; 95% CI, 0.64 to 0.87). The diagnostic sensitivity of Fries was 98% (95% CI, 0.96 to 1.0), and the diagnostic specificity was 83% (95% CI, 0.70 to 0.91). Many of the combinations of classification grounds showed similar figures, as indicated by the fact that most CIs overlapped. The theoretical combination of ‘Fries and ACR-82’ and/or ‘Fries and SLICC-12’ performed best with regard to diagnostic sensitivity and specificity. ‘ACR-82 and/or Fries’, as used in our regional register KLURING, resulted in sensitivity and specificity figures virtually similar to those for ‘ACR-82 and/or SLICC-12’ and ‘Fries and/or ACR-97’, respectively. In patients with newly diagnosed (0 to 4 years) SLE, the sensitivity appeared to be slightly better with SLICC-12 than with ACR-97, and it was obviously better than ACR-82 (Table 4).

**Table 3:** **Sensitivity, specificity and positive and negative predictive values for each separate classification ground, as well as for some combinations** ^**a**^

	**Sensitivity**	**Specificity**	**PPV**	**NPV**
Fries	0.98 (0.95 to 0.99)	0.81 (0.68 to 0.90)	0.84 (0.75 to 0.94)	0.94 (0.86 to 1.00)
ACR-82	0.80 (0.75 to 0.85)	1 (0.91 to 1.00)	1 (0.93 to 1.00)	0.81 (0.71 to 0.91)
ACR-97	0.90 (0.85 to 0.93)	0.92 (0.81 to 0.98)	0.93 (0.84 to 1.00)	0.87 (0.78 to 0.97)
SLICC-12	0.94 (0.90 to 0.96)	0.74 (0.60 to 0.84)	0.79 (0.70 to 0.89)	0.92 (0.83 to 1.00)
SLICC-12:2	0.89 (0.84 to 0.92)	0.77 (0.64 to 0.87)	0.81 (0.71 to 0.91)	0.87 (0.76 to 0.98)
Fries and/or ACR-82	0.99 (0.97 to 1.00)	0.81 (0.68 to 0.90)	0.85 (0.75 to 0.94)	0.96 (0.87 to 1.00)
Fries and/or ACR-97	1 (0.97 to 1.00)	0.81 (0.68 to 0.90)	0.85 (0.75 to 0.94)	0.96 (0.87 to 1.00)
Fries and/or SLICC-12	0.99 (0.97 to 1.00)	0.68 (0.54 to 0.79)	0.77 (0.67 to 0.86)	0.99 (0.91 to 1.00)
ACR-82 and/or SLICC-12	0.96 (0.92 to 0.98)	0.74 (0.60 to 0.84)	0.80 (0.70 to 0.89)	0.95 (0.86 to 1.00)
ACR-82 and/or SLICC-12:2	0.93 (0.90 to 0.96)	0.77 (0.64 to 0.87)	0.82 (0.72 to 0.91)	0.92 (0.82 to 1.00)
At least one of Fries, ACR-82 or SLICC-12	1 (0.98 to 1.00)	0.68 (0.54 to 0.79)	0.77 (0.67 to 0.87)	1 (0.92 to 1.00)
At least one of Fries, ACR-82 or SLICC-12:2	1 (0.98 to 1.00)	0.72 (0.58 to 0.82)	0.79 (0.70 to 0.89)	1 (0.92 to 1.00)
(Fries **+** ACR-82) and/or (Fries **+** SLICC-12)	0.94 (0.90 to 0.96)	0.87 (0.75 to 0.94)	0.88 (0.79 to 0.97)	0.90 (0.82 to 0.99)

^a^ACR-82, American College of Rheumatology 1982 criteria; ACR-97, American College of Rheumatology 1997 criteria; Fries, Fries and Holman’s diagnostic principle [7]; NPV, Negative predictive value; PPV, Positive predictive value; SLICC-12, 2012 Systemic Lupus International Collaborating Clinics criteria; SLICC-12:2, SLICC-12 criteria with a requirement for involvement of at least two organ systems for SLE diagnosis. Data are presented with 95% confidence intervals in parentheses.

**Table 4:** **Sensitivity for each separate classification ground for groups with different disease durations** ^**a**^

	**0 to 4 years (** ***n*** **= 33)**	**5 to 9 years (** ***n*** **= 53)**	**10 to 14 years (** ***n*** **= 47)**	**15 to 19 years (** ***n*** **= 43)**	**≥20 years (** ***n*** **= 69)**
Fries	0.94 (0.80 to 1.00)	0.96 (0.86 to 1.00)	0.96 (0.85 to 1.00)	0.91 (0.78 to 0.98)	0.96 (0.87 to 0.99)
ACR-82	0.60 (0.44 to 0.75)	0.89 (0.77 to 0.96)	0.76 (0.62 to 0.86)	0.69 (0.54 to 0.81)	0.86 (0.75 to 0.92)
ACR-97	0.82 (0.65 to 0.92)	0.94 (0.84 to 0.99)	0.91 (0.79 to 0.98)	0.86 (0.72 to 0.94)	0.91 (0.82 to 0.97)
SLICC-12	0.89 (0.73 to 0.97)	0.89 (0.77 to 0.96)	0.88 (0.75 to 0.95)	0.84 (0.71 to 0.93)	0.97 (0.89 to 1.00)
SLICC-12:2	0.80 (0.64 to 0.91)	0.84 (0.71 to 0.92)	0.84 (0.70 to 0.92)	0.80 (0.66 to 0.90)	0.94 (0.85 to 0.98)

^a^ACR-82, American College of Rheumatology 1982 criteria; ACR-97, American College of Rheumatology 1997 criteria; Fries, Fries and Holman’s diagnostic principle [7]; SLICC-12, 2012 Systemic Lupus International Collaborating Clinics criteria; SLICC-12:2, SLICC-12 criteria with a requirement for involvement of at least two organ systems for SLE diagnosis. Data are presented with 95% confidence intervals in parentheses. The groups are based on the 243 patients with confirmed systemic lupus erythematosus plus the 2 controls with biopsy-proven lupus nephritis.

## Discussion

Classification criteria were originally created to describe and characterize patient populations primarily in research settings with the pursuit of a high specificity for established disease [15]. Although the 1982 ACR set of classification criteria was never intended to be diagnostic, many clinicians have leaned against these as a diagnostic tool. However, as pointed out by Bertsias *et al.*, this procedure is beset with certain caveats regarding both over- and underestimation [16]. Thus, it is important to underline that disease management decisions must often be made even if the classification criteria are not strictly met.

In the present study, we aimed to evaluate the performance of SLICC-12 in a ‘real-life’ clinical setting. Hence, the SLICC-12 criteria were applied to confirmed cases in our regional SLE register, as well as to individuals who were referred to rheumatology specialists on the basis of a fair suspicion of systemic autoimmune disease, including the presence of any SLE-related autoantibody. Fulfilment of either the Fries and/or ACR-82 criteria permitted entry of these patients into the KLURING cohort, and it could thus be argued that this would favour these criteria with regard to sensitivity and specificity in this evaluation. However, in addition to also including ACR-97 in the comparisons, we believe that the present study has additional benefits. For instance, the controls did not have established rheumatological diagnoses, a situation that obviously well reflects the real-life scenario with diagnostic dilemmas. Our main findings are that (1) SLICC-12 has high diagnostic sensitivity (particularly in patients with recent-onset disease), which is in line with the results of others [10,17]; and (2) ACR-82 fails to identify every fifth true SLE case. The latter finding confirms the results of a Norwegian study based on 346 patients with connective tissue disease [18]. To our surprise, however, the disease specificity for SLICC-12 was as low as 74% and did not change much with the addition of a requirement that at least two different organ systems must be involved. SLICC-12 misclassified 14 of the 55 controls, compared to 10 using Fries, 4 using ACR-97 and none using ACR-82.

Three studies resembling our present study have recently been published. Sag *et al.* presented data from three European paediatric lupus centres regarding the performance of SLICC-12 in comparison with ACR-97 in 154 SLE cases and 123 controls in whom ANA analysis within the diagnostic workup was deemed necessary by the attending physician [19]. Control cases with established disease were selected (juvenile idiopathic arthritis was the most common diagnosis), whereas none was judged as SLE. The authors concluded that SLICC-12 was more sensitive and resulted in fewer misclassifications, whereas ACR-97 was more specific. Amezcua-Guerra *et al.* performed a chart review of 100 patients with a clinical diagnosis of SLE and 100 patients with established rheumatic disease, mainly RA [17]. They concluded that ACR-97 and SLICC-12 performed similarly well with regard to sensitivity as well as specificity. Pons-Estel *et al.* applied SLICC-12 in two multiethnic SLE cohorts and evaluated whether SLICC-12 would allow earlier classification of SLE, particularly in patients with LN, compared to ACR-97 [20]. Even though the interpretation was hampered by the fact that the two cohorts did not have similar entry criteria, the authors concluded that earlier classification of SLE with or without renal involvement was possible using SLICC-12, although it was not as clear in the LUMINA group as in the GLADEL cohort.

Contrary to the studies referred to above, the patients included in our present study were recruited from a single rheumatology unit and evaluated clinically and followed with regard to disease phenotype, activity and organ damage for a long period of time by a very limited number of rheumatology specialists [11,21]. In addition, all serological tests were analysed at the same accredited laboratory. Among the confirmed cases, 92% were Caucasians, which is fairly representative of a European SLE population [22]. The number of fulfilled ACR-82 criteria among the confirmed SLE cases (mean, 4.6) may appear low, but it is explained by the fact that our centre is the only one that diagnoses and treats adult patients with SLE in the catchment area of Linköping, Sweden. This implies that we have no bias due to selection of severe SLE cases. Thus, we can conclude that about 96% of the expected SLE cases and 99% of all known adult SLE cases were included in the study [23]. Consequently, this means that our unit takes care of the whole adult SLE spectrum, from uncomplicated cases with quiescent disease and skin and/or joint involvement to severely ill individuals with full-blown multisystemic disease.

A limitation of the study is the low number of controls. However, to compensate for this, we provide 95% CIs to indicate part of the uncertainty. The procedure of using 1 million random samples of the same size as the controls from the confirmed cases provided the best possible estimates of PPV and NPV, whereas the 95% CIs could still be based upon the appropriate number of patients (*n* = 110) to indicate the level of uncertainty (that is, wide CIs). Another possible limitation is that IgA class antibodies against PL-related antigens, which should be regarded according to SLICC-12, were not analysed. Thus, it is possible that the addition of data on IgA antibodies to cardiolipin and/or β_2_-glycoprotein-1 could have increased the SLICC-12 sensitivity figures. However, to our opinion, more careful evaluations of these IgA class antibody tests are still required before their benefits can be advocated for clinical practice [24].

SLICC-12 was elaborated in order to achieve an updated, relevant and meaningful set of SLE criteria with high sensitivity. One important distinction from ACR-82 and ACR-97 is that SLICC-12 demands at least one immunologic criterion, prohibiting the SLE diagnosis from being based solely on clinical manifestations [10]. At the same time, because this may increase the diagnostic specificity for SLE, it could also have opposite effects. For instance, and as demonstrated here, patients with APS (without SLE) commonly present with thrombocytopenia and complement consumption in combination with ANA and antibodies against PL-related antigens, and would thus incorrectly be classified as SLE according to SLICC-12 criteria. Another challenge, including several laboratory items in the classification grounds, is the choice of methods and definition of accurate cutoff levels. In our view, it is unfortunate that the SLICC group refrained from postulating that IF microscopy should remain the reference method for ANA analysis, referring to an ‘abnormal titre’ (that is, a cutoff based on a reference population) to qualify as a positive test in the new set of criteria [10].

## Conclusions

This study is the first to evaluate the performance of SLICC-12 in an adult European study population. We affirm that SLICC-12 has advantages with regard to diagnostic sensitivity in comparison with other classification grounds on the basis of our study of (1) patients in our regional register with confirmed and well-characterized SLE and (2) autoantibody-positive patients who had been referred to rheumatology specialists on the basis of a fair suspicion of systemic autoimmune disease. However, whereas the sensitivity appeared to be superior with SLICC-12 compared with ACR-82 and ACR-97 (particularly in newly diagnosed cases), the diagnostic specificity for SLICC-12 was surprisingly low. Thus, to realise the greatest sensitivity and specificity, a combination of criteria for clinical SLE studies should be considered.

## Abbreviations

ACR: American College of Rheumatology
ANA: Antinuclear antibody
anti-dsDNA: Antibodies to double-stranded DNA
APS: Antiphospholipid syndrome
CI: Confidence interval
ENA: Extractable nuclear antigen
FITC: Fluorescein isothiocyanate
IF: Immunofluorescence
Ig: Immunoglobulin
KLURING: Clinical Lupus Register in Northeastern Gothia
LN: Lupus nephritis
NPV: Negative predictive value
PL: Phospholipid
PPV: Positive predictive value
RA: Rheumatoid arthritis
SLE: Systemic lupus erythematosus
SLICC: Systemic Lupus International Collaborating Clinics
UCTD: Undifferentiated connective tissue disease
